# Application and Performance of Artificial Intelligence Technology in Detection, Diagnosis and Prediction of Dental Caries (DC)—A Systematic Review

**DOI:** 10.3390/diagnostics12051083

**Published:** 2022-04-26

**Authors:** Sanjeev B. Khanagar, Khalid Alfouzan, Mohammed Awawdeh, Lubna Alkadi, Farraj Albalawi, Abdulmohsen Alfadley

**Affiliations:** 1 Preventive Dental Science Department, College of Dentistry, King Saud bin Abdulaziz University for Health Sciences, Riyadh 11426, Saudi Arabia; m97a97@gmail.com (M.A.); balawif@ksau-hs.edu.sa (F.A.); 2 King Abdullah International Medical Research Centre, Ministry of National Guard Health Affairs, Riyadh 11481, Saudi Arabia; kalfouzan@yahoo.com (K.A.); lubna.alkadi@gmail.com (L.A.); fadleya@ksau-hs.edu.sa (A.A.); 3 Restorative and Prosthetic Dental Sciences Department, College of Dentistry, King Saud bin Abdulaziz University for Health Sciences, Riyadh 11426, Saudi Arabia

**Keywords:** artificial intelligence, dental caries, diagnosis, detection, prediction

## Abstract

Evolution in the fields of science and technology has led to the development of newer applications based on Artificial Intelligence (AI) technology that have been widely used in medical sciences. AI-technology has been employed in a wide range of applications related to the diagnosis of oral diseases that have demonstrated phenomenal precision and accuracy in their performance. The aim of this systematic review is to report on the diagnostic accuracy and performance of AI-based models designed for detection, diagnosis, and prediction of dental caries (DC). Eminent electronic databases (PubMed, Google scholar, Scopus, Web of science, Embase, Cochrane, Saudi Digital Library) were searched for relevant articles that were published from January 2000 until February 2022. A total of 34 articles that met the selection criteria were critically analyzed based on QUADAS-2 guidelines. The certainty of the evidence of the included studies was assessed using the GRADE approach. AI has been widely applied for prediction of DC, for detection and diagnosis of DC and for classification of DC. These models have demonstrated excellent performance and can be used in clinical practice for enhancing the diagnostic performance, treatment quality and patient outcome and can also be applied to identify patients with a higher risk of developing DC.

## 1. Introduction

Oral diseases like dental caries (DC) and periodontal diseases pose a major disease burden and are considered non-fatal causes of disability affecting people of all age groups globally [1]. The pain and discomfort that is often associated with DC may eventually compromise the individual’s sleep, diet, social well-being and self-esteem which can affect quality of life [2]. According to the global burden of disease, untreated DC are the most prevalent and common factor affecting health [1]. On the global scale, it is estimated that DC are prevalent among 2.3 billion adults with secondary dentition, and among 530 million children with deciduous dentition [3]. 

In 2015, the global cost of oral diseases was reported to exceed 540 billion dollars, consequently leading to major health and financial burden [4]. Early and accurate detection of DC can enable cost effective preventive measures and more conservative treatment options, reducing the healthcare costs [5]. Traditionally, visual inspection in combination with radiographic assessment is the routine diagnostic approach for DC. However, studies indicated the presence of considerable variability in its reliability and accuracy, affected mainly by the level of dentists’ clinical experience. Sensitivity can range between 19–92% for occlusal and 39–94% for proximal DC [6]. Various parameters like shadow, contrast, and brightness in radiographs may have an impact on the diagnosis [7].

The recent advancements in techniques used for the detection and diagnosis of DC resulted in the development of novel methods that aim to overcome the constraints of clinical and radiographic diagnosis. These include ultrasonic detection of caries, laser fluorescence, digital imaging fiber-optic trans-illumination (FOTI), quantitative light-induced fluorescence (QLF), digital subtraction radiography (DSR), tuned aperture computed tomography (TACT), and electrical conductance measurement (ECM) [8,9]. Laser fluorescence has relatively higher sensitivity in diagnosing early DC in comparison with other methods [10]. However, studies have reported on the limitations of these techniques; FOTI demonstrates low sensitivity when used for the diagnosis of proximal DC [11] and ultrasonic devices are only capable of detecting established DC [12]. Caries risk prediction models like the caries-risk assessment tool (CAT), caries management by risk assessment (CAMBRA), and Cariogram are commonly used for predicting DC. Nevertheless, these models lack sufficient evidence to prove their effectiveness. A systematic review reported that the sensitivity and specificity of Cariogram ranged between 41–75% and 65.8–88%, respectively [13]. 

With the present evolution in the fields of science and technology, newer applications based on artificial intelligence (AI) technology have been widely used in medical sciences. These models have demonstrated excellent performance and high accuracy and sensitivity in performing their intended tasks, including diagnosing eye disorders, and breast and skin cancers, detection and diagnosis of pulmonary nodules [14,15,16,17]. AI models have also been widely applied in detection, segmentation and classification of coronavirus disease of 2019 (COVID-19) using computerized tomography (CT) medical images and these models have demonstrated substantial potential in rapid diagnosis of COVID-19 [18]. Hence, with a growing interest in these AI-based applications, these models have now been employed in a wide range of applications related to the diagnosis of oral diseases and have demonstrated phenomenal precision and accuracy in their performance [19,20,21,22]. Studies have reported on the application and performance of the AI models in various disciplines of dentistry, which includes orthodontics, restorative dentistry and prosthodontics [23,24,25]. However, there are no systematic review articles exclusively reporting on application of AI models on dental caries. Additionally, detecting DC using AI-based models has been found to be a cost-effective approach, where the AI-based DC detection model demonstrated higher accuracy in detecting DC in comparison with trained examiners, with fewer chances of false negative errors [26]. 

Hence, the aim of this systematic review is to report on the diagnostic accuracy and performance of AI-based modes designed for detection, diagnosis, and prediction of DC.

## 2. Materials and Methods

### 2.1. Search Strategy

This systematic review was executed in compliance with the standards of preferred reporting items for systematic reviews and meta-analysis for diagnostic test accuracy (PRISMA-DTA) [27]. The literature search for this paper was based on the population, intervention, comparison, and outcome (PICO) criteria (Table 1).

Eminent electronic databases (PubMed, Google scholar, Scopus, Web of science, Embase, Cochrane, Saudi Digital Library) were searched for relevant articles that were published from January 2000 until February 2022. The literature search was based on the Medical Subject Headings (MeSH) terms like dental caries, tooth decay, cavity, diagnosis, detection, prediction, artificial intelligence, machine learning, deep learning (DL), automated system, convolutional neural networks (CNNs), artificial neural networks (ANNs) and deep neural networks (DCNNs). A combination of these MeSH terms using Boolean operators and/or were used in the advanced search for the articles. Manual searches for additional articles was also carried out in the college library based on the reference lists extracted from the initially selected articles

### 2.2. Study Selection

Article selection was conducted in two phases. During the initial phase, articles were selected based on the relevance of the title and abstract to the research question. In this phase, the article search was done by two authors (S.B.K and F.B) independently, and this process generated 448 articles. These articles were further screened to eliminate the duplicates, ultimately leading to the exclusion of 168 articles. The remaining 280 articles were evaluated based on the eligibility criteria. 

### 2.3. Eligibility Criteria

In this systematic review, the articles were included based on being (a) Original research articles reporting on application of AI-based models in diagnosis, detection and prediction of DC, (b) Articles reporting on the data sets used for training/validating and testing of the model, (c) Articles with clear information on quantifiable performance outcome measures, (d) The type of study design did not limit its inclusion. 

The articles excluded were (a) Articles with only abstracts, without full text availability, (b) Conference proceedings, commentaries, editorial letters, short communications, review articles and scientific posters uploaded online, (c) Articles published in non-English languages.

### 2.4. Data Extraction

**Figure 1:**
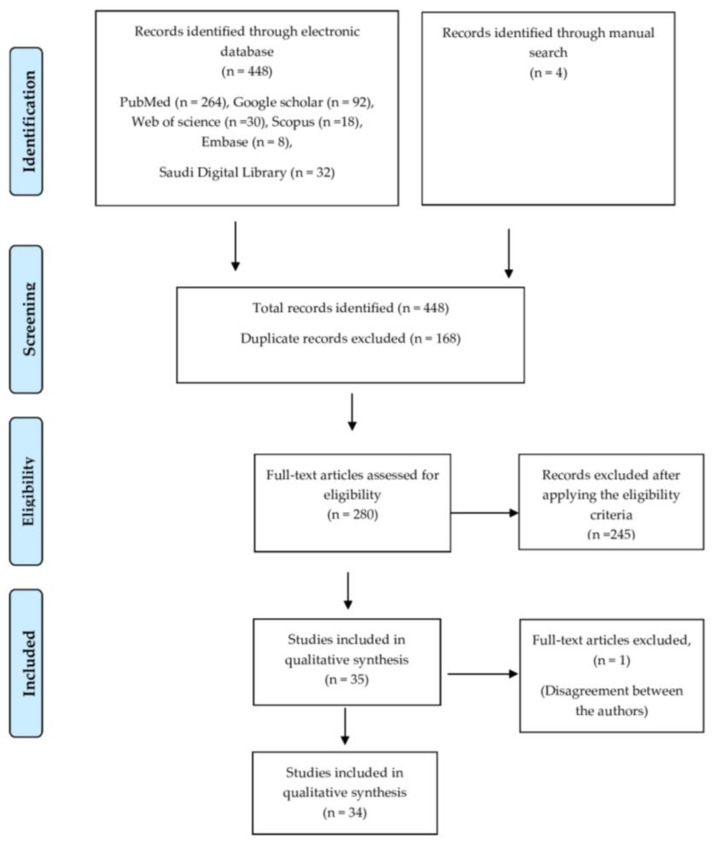
Flow chart for screening and selection of articles.

280 articles obtained from the initial phase were further evaluated based on these eligibility criteria. Following this, the number of articles decreased to 35. In the final phase, the authors’ details and information were concealed and the articles were assigned for further analysis by two authors (M.A and L.A), who were not involved in the initial phase of evaluation. In order to determine the degree of consistency between these two authors, inter-rater reliability was assessed. Cohen’s kappa showed 84% agreement between these authors. These articles were critically analyzed based on the guidelines of quality assessment and diagnostic accuracy tool (QUADAS-2) [28]. This is a revised tool used for assessing the quality of studies that have reported on diagnostic tools. Quality assessment was carried out based on four main domains (patient selection, index test, reference standard, and flow and timing), which were evaluated for risk of bias and applicability concerns [28]. The authors further had contrasting opinions about the inclusion of one article that did not clearly mention the outcome measures. The issue was resolved upon discussion with another author (A.F), and a decision to exclude the article was made. A total of 34 articles were subjected to quantitative synthesis (Figure 1).

## 3. Results

Thirty-four articles [29,30,31,32,33,34,35,36,37,38,39,40,41,42,43,44,45,46,47,48,49,50,51,52,53,54,55,56,57,58,59,60,61,62] that met the selection criteria were assessed for quantitative data (Table 2). The research trend shows that most of the research on application of AI on DC was conducted within the last few years and the trend shows a gradual increase in this area of research.

### 3.1. Qualitative Synthesis of the Included Studies

AI has been applied for prediction of DC (n = 8) [29,33,40,45,46,53,55,57], for detection and diagnosis of DC (n = 24) [30,31,32,34,35,36,38,39,41,42,43,44,47,48,49,50,52,56,58,59,60,61,62], and for classification of DC (n = 2) [31,37,45,51]. The data from selected articles were retrieved and entered into the data sheet.

With this data, performing a meta-analysis was not possible due to the heterogeneity between the studies in the software and data sets used for assessment of performance of the AI models. Therefore, the descriptive data was presented based on the application of AI models for which it has been designed.

### 3.2. Study Characteristics

The data mainly included details of the study (details of authors, publication year, type of algorithm architecture used, study objective, number of patients/images/photographs/radiographs for validating and testing, study factor, study modality, comparisons, evaluation accuracy/average accuracy/statistical significance, outcomes and conclusions).

### 3.3. Outcome Measures

The outcome was measured in terms of task performance efficiency. The outcome measures were reported in terms of accuracy, sensitivity, specificity, ROC = receiver operating characteristic curve, AUC = area under the curve, AUROC = area under the receiver operating characteristic, ICC = intraclass correlation coefficient, IOU = intersection-over-union, PRC = precision recall curve, statistical significance, F1 Scores, vDSC = volumetric dice similarity coefficient, sDSC = surface dice similarity coefficient, PPV = positive predictive value, NPV = negative predictive value, MDG = mean decreased gini, MDA = mean decreased accuracy coefficients, IoU = intersection over union, dice coefficient [23,24,25,26,27,28,29,30,31,32,33,34,35,36,37,38,39,40,41,42,43,44,45,46,47,48,49,50,51,52,53,54,55,56].

### 3.4. Risk of Bias Assessment and Applicability Concerns

**Figure 2:**
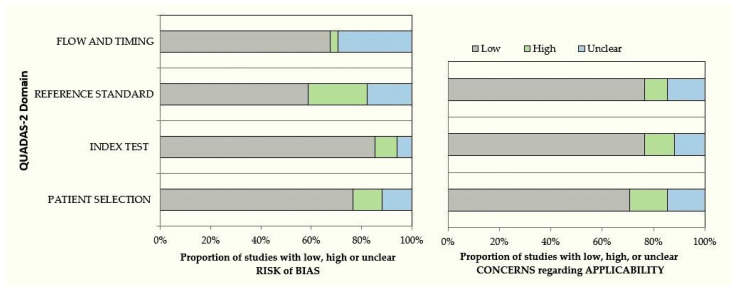
QUADAS-2 assessment of the individual risk of bias domains and applicability concerns.

The quality assessment of the 18 articles included in this study was done using the guidelines of QUADAS-2 [13]. This tool was originally produced in 2003 by collaboration between the Centre for Reviews and Dissemination, University of York, and the Academic Medical Centre at the University of Amsterdam. Modified versions have been adopted by Cochrane Collaboration, NICE and AHRQ. The current version is widely used in systematic reviews to evaluate the risk of bias and applicability of primary diagnostic accuracy studies. QUADAS-2, consists of four key domains: patient selection; index test; reference standard; flow and timing. The current assessment of risk and applicability based on QUADAS-2 shows that the majority of studies have low risk and a very small number of studies show high risk of bias. (Supplementary Table S1) (Figure 2).

### 3.5. Assessment of Strength of Evidence 

The articles included in this systematic review were assessed for the certainty of the evidence using the grading of recommendations assessment, development and evaluation (GRADE) approach. The certainty of evidence is rated based on five domains: risk of bias, inconsistency, indirectness, imprecision, or publication bias and are ultimately categorized as either very low, low, moderate, or high certainty of evidence [63] (Table 3).

## 4. Discussion

Oral diseases like DC and periodontal diseases are some of the major public health issues affecting people of all age groups in developing and developed countries. In most cases, DC remain undiagnosed because of deep fissures and tight interproximal contacts, making them difficult to be detected in the early stages, eventually leading to their detection in the advanced stages. Early detection of DC reduces the disease burden and need for invasive treatment procedures which can ultimately improve treatment outcome [32]. Clinical oral examination using a dental probe/explorer along with radiographs is regarded as the most conventional method in detecting DC. However, studies have also reported on the variations in accuracy and reliability among clinicians using this method, influenced by their clinical experience [7,64,65].

Automated decision support systems based on AI technology are new developments in the field of medical sciences. AI-based models have also been widely applied in the field of dentistry and have demonstrated exceptional performance in tooth detection, tooth numbering, diagnosing and predicting oral cancer, periodontal diseases, and root fractures, orthodontic diagnosis, and detection of jaw lesions, cysts and tumors [19,20,21,22]. Considering the challenges and limitations dentists face in detecting DC during clinical examination, there is a need for developing AI-based automated models that can assist dentists in decision making, increasing the accuracy of DC detection and diagnosis.

Several factors influence the risk of developing DC like oral hygiene practices, dietary habits, socio-economic status, utilization of dental care services, in addition to attitude towards oral health [66]. Hence, identifying the factors that determine the risk of developing DC in an individual is essential for its prevention. AI-based models have been widely applied for prediction of DC. Zanella-Calzada et al. [29] reported on an AI-based model for analyzing the dietary and demographic factors that determine DC using data sets, where the model demonstrated an accuracy of 0.69 and AUC values of 0.69 and 0.75. This model showed good accuracy for classifying individuals with and without caries based on dietary and demographic factors. The main advantage of this model is that the data used for training the model was obtained from subjects from different regions, hence providing robustness in results, and eliminating the bias to subject selection. Hung M et al. [33] proposed an AI-based ML model for diagnostic prediction of root caries. This model demonstrated an excellent performance with an accuracy of 97.1%, a precision of 95.1%, the sensitivity of 99.6%, the specificity of 94.3% and an AUC of 0.997. Although the model demonstrated excellent performance, there were certain limitations related to the data sets used for its development. In this model, the data was obtained from a sample of the United States (US) population, and therefore, would be more representative of the US nationals and not of patients with different demographic data. Another important limitation was that the authors did not consider some important covariates like lifestyle and oral hygiene factors.

Ramos-Gomez et al. [40] described an AI-based ML algorithm (Random Forest) for identifying survey items that predict DC. The model demonstrated a mean decreased Gini coefficient (MDG) of 0.84; and a mean decreased accuracy (MDA) of 1.97 for classifying active DC based on parent’s age. For predicting DC based on parents age, the model demonstrated an MDG = 2.97; MDA = 4.74. This model can be of potential use for screening children for DC based on the survey data. The study had several limitations which include the limited sample size for testing obtained from limited hospital records that are not representative of the general population. In addition, the data regarding children’s oral hygiene practices were obtained from their parents, giving rise to social desirability bias. Zaorska et al. [45] reported on an AI model for predicting DC based on chosen polymorphisms. The model demonstrated a sensitivity of 90%, a specificity of 96%, an overall accuracy of 93% (*p* < 0.0001) and the AUC was 0.970 (*p* < 0.0001). Prediction accuracy of 90.9–98.4% was achieved by this model. The main strength of this model was the homogeneous age and gender of study subjects, and that the assessment of performance was carried out using two different statistical approaches, rendering results that are more reliable. However, the sample used for validating the model was limited. Pang et al. [46] reported on an AI-based ML model for caries risk prediction based on environmental and genetic factors. The model demonstrated an AUC of 0.73. This model could accurately identify individuals at high and very high caries risk. However, the sample was confined to only one center and early signs of DC were not detected in this study. In a study by Hur et al. [53] an ML model for predicting DC on second molars associated with impacted third molars was tested. This model demonstrated good accuracy with a ROC of 0.88 to 0.89. However, the authors did not consider the major DC contributing factors like oral hygiene and dietary intake of sugars. Park et al. [57] also reported on an ML model for predicting early childhood caries. The model demonstrated a favorable performance with an AUROC of 0.774–0.785. Limitations of this study included low specificity values and potential bias resulting from the consideration of maternal variables solely. Additionally, the authors did not consider important variables like feeding practices, sugar intake and usage of fluoride.

Undiagnosed and untreated DC are major public health problems affecting billions of people worldwide. Early detection of DC can significantly reduce the need for invasive treatment and ultimately the cost of care. Hence, diagnostic tools with high accuracy in detecting DC are needed. Lee et al. [30] reported on a DL model for detecting and diagnosing DC on periapical radiographs. The model demonstrated an accuracy of 89.0%, 88.0%, 82.0% and AUC of 0.917 for premolar, molar, and both premolar and molar models respectively. DL model CapsNet is a recently developed model made of deep players and is very effective for processing visual factors from posture, speed, hue, and texture [67]. Therefore, models of this nature are enabled with improved and optimized features for the detection and diagnosis of DC [68]. Although the model demonstrated considerable performance, there were limitations related to unconsidered clinical parameters, limited number of radiographs, and the inclusion of permanent teeth only [30]. Choi et al. [31] reported on an automated model for the detection of proximal DC in periapical radiographs. The proposed model was found to be superior to the system using naïve CNNs. Casalegno et al. [32] reported on a DL model for the automated detection and localization of DC. This model demonstrated promising results with increased speed and accuracy in detecting DC. However, the model had some shortcomings, where physically unrealistic labeling of artifacts took place, especially in underexposed and overexposed areas. Cantu et al. [34] proposed a DL model for detecting DC on bitewing radiographs. The model demonstrated an accuracy of 0.80; sensitivity of 0.75 and specificity of 0.83. The accuracy of this model was higher than that of experienced dentists in detecting initial lesions. The main strength of this study was the large number of balanced data sets used in training and testing. These results were similar to the results of another study conducted by Lee et al. [52] on a deep CNNs (U-Net) model for detection of DC on bitewing radiographs, where the model demonstrated a precision of 63.29%, a recall of 65.02%, and an F1-score of 64.14%. However, the limitation of this study was related to the small number of data sets. Geetha et al. [35] reported on an AI-based model for diagnosing DC on digital radiographs. The model showed excellent performance with an accuracy of 97.1%, a false positive (FP) rate of 2.8% and a ROC area of 0.987. However, the model must be improved to enable classification of DC based on lesion depth.

Another study conducted by Schwendicke et al. [36] also reported on an AI-based model for detecting DC. The performance of this model was comparable with that of trained dentists. The limitation of this study was related to the reliability of the examiners. Duong et al. [37] also reported on an AI-based model for detecting DC using photographs on smart phones. The model demonstrated an accuracy of 92.37%, a sensitivity of 88.1% and a specificity of 96.6%. However, there was no heterogeneity in the data sets and the presence of plaque, debris, stains and shadow may have affected the results. A study conducted by Zhang et al. [60] suggested a CNNs-based model (ConvNet) for detecting DC using oral photographs. The model demonstrated an AUC of 85.65% and a sensitivity of 81.90%. However, the dataset was collected from a single organization, which can limit its applicability. In addition, factors like the presence of plaque and stains could have affected the obtained results. Another study conducted by Kühnisch et al. [61] reported on a CNNs-based model for detection and categorization of DC using oral photographs. In this study, the model demonstrated an excellent accuracy of 92.5%, a sensitivity of 89.6%, and a specificity of 94.3%. This study had taken into consideration the limitations of the previously reported studies and therefore only considered photographs that were free from plaque, calculus and saliva.

A study by Devlin et al. [43] proposed an AI-based model for detecting enamel-only proximal DC using bitewing radiographs. The model demonstrated significant results in comparison with expert dentists. Bayrakdar et al. [44] also reported on AI-based DL models (VGG-16 and U-Net) for automatic caries detection and segmentation on bitewing radiographs. These models demonstrated superior performance in comparison with experienced specialists. However, this study was compromised by the limited data sets obtained from one center. Zheng et al. [47] compared three CNNs models (VGG19, Inception V3, and ResNet18) for diagnosing deep DC. CNNs model ResNet18 showed good performance in comparison with the other two models and the trained dentists. However, diagnosis of cases was done by a panel of experienced dentists, which is not the gold standard for diagnosing deep DC and pulpitis. Nevertheless, histological testing, which is the gold standard, is not practically feasible in clinical practice [69]. Another study conducted by Moran et al. [49] reported on a CNNs model (Inception) for identifying approximal DC on bitewing radiographs. The model demonstrated an accuracy of 73.3%. This model demonstrated promising results in comparison with the reference model (ResNet). Mertens et al. [50] reported on a CNNs model for the detection of proximal DC using bitewing radiographs. The model demonstrated a ROC of 0.89 and a sensitivity of 0.81 and showed significant results in comparison with five expert dentists. The main strength of this study was its design being a randomized controlled trial. On the other hand, the main drawback was related to the limited sample of data sets which were obtained from one center. Another study conducted by Mao et al. [56] used a CNNs-based model for identifying DC on bitewing radiographs. The model demonstrated an accuracy of 90.30% for detecting DC. The AlexNet model showed a high accuracy in comparison with other models. To achieve better accuracy, the authors had reduced the size of photographs used in the training process, which reduced training time and increased the accuracy of the model. A study conducted by Bayraktar et al. [59] described a CNNs based model (YOLO) for the diagnosis of interproximal caries lesions on bitewing radiographs. The model demonstrated an excellent accuracy of 94.59%, a sensitivity of 72.26%, and a specificity of 98.19%. The main strength of this study was the large number of data sets which yielded near perfect results. However, the model could not classify the DC lesions according to their location in enamel and/or dentin.

Lian et al. [48] also reported on DL models for detecting DC and classifying DC on panoramic radiographs. The models demonstrated Dice coefficient values of 0.663 and an accuracy of 0.986. Their performance was similar to that of expert dentists. The strength of this study was the large data which was meticulously collected and labeled by three expert dentists. Controversial results were additionally revised by a fourth expert dentist. However, the panoramic radiographs used in this study were obtained from one single machine, hence, the performance may vary with panoramic radiographs obtained using equipment from another company. A study conducted by De Araujo Faria et al. [54] reported on an AI-based model for the prediction and detection of radiation-related caries (RRC) on panoramic radiographs. This model demonstrated an excellent detection accuracy of 98.8% and an AUC of 0.9869. For prediction, it showed an accuracy of 99.2% and an AUC of 0.9886. However, the limited sample size may have affected the results, as the patients in that particular center were usually at an advanced DC stage by the time the radiographs were obtained. Zhu et al. [62] also reported on a CNNs-based model (CariesNet) to delineate different degrees of caries on panoramic radiographs. The model demonstrated an excellent performance with a mean dice coefficient of 93.64%, an accuracy of 93.61%, an F1 score of 92.87% and a precision of 94.09%. The large number of datasets used to train and validate the model was a strength of this study.

Huang et al. [68] reported on AI-based models AlexNet, VGG-16, ResNet-152, Xception, and ResNext-101 for detecting DC on OCT and micro-CT images. ResNet-152 demonstrated the highest accuracy rate of 95.21% and a sensitivity of 98.85% in comparison with the other three models. However, the study utilized a manual verification process, where human errors are inevitable.

## 5. Conclusions

AI models have been widely explored for prediction, detection and diagnosis of DC. These models have demonstrated excellent performance and can be used in clinical practice for identifying patients with higher DC risk and can also aid in enhancing the diagnostic, treatment quality and patient outcome. The results of the predictive models can help in planning preventive dental care, designing oral hygiene care and dietary plans for patients with high risk of DC. These models can assist dentists as a supportive tool in clinical practice and can also assist non-dental professionals in detecting and diagnosing DC more accurately in schools and rural health centers. Although these models have demonstrated excellent performance, there are certain limitations related to the size and heterogeneity of the data sets reported in most of these articles. Hence, these models need additional training and validation for better performance.

## Figures and Tables

**Table 1:** Description of the PICO (P = Population, I = Intervention, C = Comparison, O = Outcome) elements.

**Research Question**	What is the performance of AI-based models designed for detection, diagnosis and prediction of DC?
**Population**	Patients who underwent investigation for DC
**Intervention**	AI applications for detection, diagnosis and prediction of DC
**Comparison**	Expert/Specialist opinions, Reference standards/models
**Outcome**	Measurable or predictive outcomes such as accuracy, sensitivity, specificity, ROC = receiver operating characteristic curve, AUC = area under the curve, AUROC = area under the receiver operating characteristic, ICC = intraclass correlation coefficient, IOU = intersection-over-union, PRC = precision recall curve, statistical significance, F1 scores, vDSC = volumetric dice similarity coefficient, sDSC = surface dice similarity coefficient, PPV= positive predictive value, NPV = negative predictive value, mean decreased gini (MDG), mean decreased accuracy (MDA) coefficients, intersection over union (IoU), dice coefficient

**Table 2:** Details of the studies that have used AI-based models for detection, diagnosis and prediction of DC.

Serial No.	Authors	Year of Publication	Study Design	Algorithm Architecture	Objective of the Study	No. of Patients/Images/Photographs for Testing	Study Factor	Modality	Comparison if Any	Evaluation Accuracy/Average Accuracy/Statistical Significance	Results (+)Effective, (−)Non Effective (N) Neutral	Outcomes	Authors Suggestions/Conclusions
1	Zanella-Calzada et al. [29]	2018	Prospective cohort	ANNs	To analyze the dietary and demographic factors that determine oral health and DC	6868 cases for training, 2944 cases for testing	DC lesions	Data sets	National Health and Nutrition Examination Survey Data	Accuracy of 0.69, AUC values of 0.69 and 0.75	(+)Effective	This ANNs-based model demonstrated high accuracy in diagnosing DC based on dietary and demographic factors	This model can help dentists by providing an easy, free and fast tool for the diagnosis of DC
2	Lee et al. [30]	2018	Retrospective cohort	DCNNs	Deep CNN algorithms (GoogLeNet Inception v3) for detection and diagnosis of DC on periapical radiographs	2400 periapical radiographs for training, 600 periapical radiographs for testing	Gray/white matter and lesions	Periapical radiographs	Not mentioned	Accuracy of 89.0%, 88.0%, 82.0% and AUC of 0.917, 0.890, 0.845 for premolar, molar, and both premolar and molar models respectively.	(+)Effective	This CNNs-based model demonstrated good performance in detecting DC	This models is of potential use for detection and diagnosis of DC
3	Choi et al. [31]	2018	Retrospective cohort	CNNs	An automatic model for detection of proximal DC in periapical radiographs	475 periapical radiographs	DC lesions	Periapical radiographs	Experts and naïve CNN approach as reference models	*F*1*max* 0.74 with False Positives 0.88	(+)Effective	This model was superior to the system using a naïve CNN.	This model was successful in detecting proximal DC
4	Casalegno et al. [32]	2019	Retrospective cohort	CNNs	DL model for the automated detection and localization of DC in near-infrared transillumination (TI) images	217 grayscale images 185 for training	DC lesions	TI images	Reference deep neural networks models and experts	Mean intersection-over-union (IOU) score of 72.7% on a 5-class segmentation task and IOU score of 49.5% and 49.0% and ROC curve of 83.6% and 85.6% for proximal and occlusal carious lesions, respectively	(+)Effective	This DL approach holds promising results for increasing the speed and accuracy of caries’ detection	This model can support dentists by providing high-throughput diagnostic assistance and improving patient outcomes
5	Hung et al. [33]	2019	Retrospective cohort	ANNs	ML model for diagnostic prediction of root caries	7272 cases for training and 1818 for testing	Variables	Data sets	Trained dental professionals and reference models	Accuracy of 97.1%, precision of 95.1%, sensitivity of 99.6%, specificity of 94.3% and AUC of 0.997	(+)Effective	This model demonstrated the best performance	This model can be implemented for clinical diagnosis and can be utilized by dental and non-dental professionals
6	Cantu et al. [34]	2020	Retrospective cohort	CNNs	To assess the performance pf a DL model for detecting DC on bitewing radiographs	3293 Bitewing radiographs for training and 252 for testing	DC lesions	Bitewing radiographs	4 experienced dentists	Accuracy of 0.80; sensitivity of 0.75, specificity of 0.83	(+)Effective	This CNN-based model was significantly more accurate than the experienced dentists.	This model can assist dentists particularly in the detection of initial caries lesions on bitewings
7	Geetha et al. [35]	2020	Cross sectional	ANNs	ANN based model for diagnosing DC in digital radiographs	145 digital radiographs	DC lesions	Intraoral digital images (digital radiographs)	Experienced dentist	Accuracy of 97.1%, false positive (FP) rate of 2.8%, ROC area of 0.987 and PRC area of 0.987	(+)Effective	This model based on back-propagation neural network can predict DC more accurately	Improved algorithms and high quantity and quality datasets may demonstrate better results in clinical dental practice
8	Schwendicke et al. [36]	2020	Cross sectional	CNNs	CNN based model for detecting DC in near-infrared-light transillumination (NILT) images.	226 extracted teeth images	DC lesions	NILT images	2 experienced dentists	Mean AUC was 0.74. Sensitivity of 0.59, specificity of 0.76, PPV was 0.63, NPV 0.73	(+)Effective	These models (Resnet18 and Resnext50) showed satisfying results in detecting DC	These models can be of relevance in settings, like schools, care homes or rural outpost centers
9	Karhade D.S et al. [37]	2021	Retrospective cohort	ANNs	Automated MLalgorithm for classification early childhood caries (ECC)	6040 (5123 subjects for training 1281 subjects for testing)	Variables	Data sets	External National Health and Nutrition Examination Survey (NHANES) dataset/ 10 trained and calibrated clinical examiners	AUC of (0.74), Sensitivity of (0.67), and PPV of (0.64)	(+)Effective	This ML model’s performance was similar to the reference model	This model is valuable for ECC screening
10	Duong et al. [38]	2021	Cross sectional	ANNs	An automated ML for detecting DC using smart phone photographs	620 teeth (80% for validating and 20%for testing)	DC lesions	Photos using smartphone	4 trained and calibrated dentists	Accuracy of 92.37%, sensitivity 88.1% and specificity of 96.6% best model were and for “Cavitated versus (visually non-cavitated(VNC) + no Surface change(NSC)), whereas for “VNC versus NSC they were 83.33%, 82.2%, and 66.7% respectively	(+)Effective	This model demonstrated an auspicious potential for clinical diagnostics with reasonable accuracy and minimal cost	This support vector machine requires further improvement and verification
11	Duong et al. [39]	2021	Cross sectional	CNNs	AI based model for detection and classification of DC using smart phone photographs	587 extracted teeth 80% for training, 10% for validating 10% for testing	DC lesions	Photos using smartphone	Trained dentists	Accuracy of 87.39%, sensitivity of 89.88%, and specificity of 68.86%	(+)Effective	This model demonstrated good accuracy in the detection of DC. GoogleNet performed better than ResNet18 and ResNet50	This model needs to be trained with both in vivo and vitro images. There is a need for developing a good imaging technique for occlusal surfaces
12	Ramos-Gomez et al. [40]	2021	Retrospective cohort	ANNs	ML algorithm (Random forest) for identifying survey items that predict DC	182 subjects	Variables	Data sets	2 trained dentists	For classifying active caries parent’s age (MDG = 0.84; MDA = 1.97), unmet needs (MDG = 0.71; MDA = 2.06). Predictors of caries with parent’s age (MDG = 2.97; MDA = 4.74), with oral health problems in past 12 months (MDG = 2.20; MDA = 4.04	(+)Effective	This model showed potential for screening DC	This model showed potential for screening for DC among children using survey answers
13	Askar et al. [41]	2021	Cross sectional	CNNs	DL model for detecting white spot lesions using digital camera photographs	51 patients 2781 labelled teeth	White spot Lesions	Digital camera images	Trained dentist	Detecting any lesions (PPV/NPV) between 0.77–0.80. For detecting fluorotic lesions 0.67 (PPV) to 0.86 (NPV). For detecting other-than-fluorotic lesions 0.46 (PPV) to 0.93 (NPV).	(+)Effective	This model showed satisfying accuracy to detect white spot lesions, particularly fluorosis	There is a need for more data sets for generalizability
14	Chen et al. [42]	2021	Retrospective cohort	CNNs	DL model for detecting dental disease on periapical radiographs	2900	DC/ Periodontal disease (PDL)	Digital periapical radiographs	Reference models/trained experts	DC and PDL were detected with precision, recall, and average precision values less than 0.25 for mild level, 0.2–0.3 for moderate level and 0.5–0.6 for severe level Lesions were generally detected with precision and recall between 0.5–0.6 at all levels	(+)Effective	These model can detect DC using periapical radiographs	These models are best utilized for the detection of lesions with severe levels. Hence the models need more training at each level
15	Devlin et al. [43]	2021	Randomized control trial	CNNs	To detect enamel-only proximal DC using AssistDent artificial intelligence (AI) software on bitewing radiographs	24 patients	DC lesions	Bitewing radiographs	6 dental specialists (for grading) 23 dentists as comparison	High accuracy of diagnosis with sensitivity of 71% and decrease in specificity of 11% are statistically significant (*p* < 0.01) in comparison with expert dentists	(+)Effective	This model significantly improved dentists’ ability to detect enamel-only proximal caries	Can be used as a supportive tool by dentist to practice preventive dentistry
16	Bayrakdar et al. [44]	2021	Retrospective cohort	CNNs	DL models (VGG-16 and U-Net) for automatic caries detection and segmentation on bitewing radiographs	621 patients (2325 images, 2072 for training, 200 for validating and 53 for testing)	DC lesions	Bitewing radiographs	5 experienced experienced observers	For caries detection sensitivity 0.84, precision 0.81, and F-measure rates 0.84 and for caries segmentation were sensitivity 0.86, precision 0.84, and F-measure rates 0.84	(+)Effective	These models can accurately detect DC. There were also beneficial in the segmentation of DC	The performance of these models was superior to specialists and can be beneficial for clinicians in clinical decision making
17	Zaorska et al. [45]	2021	Prospective cohort	CNNs	AI model for predicting DC based on chosen polymorphisms	95 patients	DC lesions	Data sets	Logistic regression model	Sensitivity of 90, specificity of 96% overall accuracy of 93% (*p* < 0.0001), AUC was 0.970 (*p* < 0.0001). Prediction accuracy of 90.9–98.4%	(+)Effective	This model displayed high accuracy in predicting DC	The knowledge of potential risk status could be useful in designing oral hygiene practices and recommending dietery habits for patients
18	Pang et al. [46]	2021	Prospective cohort	ANNs	AI based ML model for caries risk prediction based on environmental and genetic factors	953 patients (633 for training and 320 for testing)	DC lesions	Data sets	Logistic regression model	AUC of 0.73	(+)Effective	This model could accurately identify individuals at high and very high caries risk	This is a powerful tool for identifying individuals at high caries risk at community-level
19	Zheng et al. [47]	2021	Cross sectional	CNNs	To evaluate and compare three CNNs models (VGG19, Inception V3, and ResNet18) for diagnosing deep DC.	844 (717 for training and 127 for testing)	Deep DC lesions	Radiographs	VGG19, Inception V3, experienced dentists	Accuracy = 0.82, precision = 0.81, sensitivity = 0.85 specificity = 0.82, AUC = 0.89,	(+)Effective	CNN model ResNet18 showed good performance	With clinical parameters this model demonstrated enhanced performance
20	Lian et al. [48]	2021	Cross sectional	CNNs	To evaluate DL methods for detecting DC lesions (nnU-Net) and classifying DC (DenseNet121) on panoramic radiographs	1160 (1071 for training and validating, 89 for testing)	DC lesions	Panoramic radiographs	6 expert expert dentists	IoU 0.785, Dice coefficient values of 0.663. Accuracy of 0.986 recall rate of 0.821	(+)Effective	These models displayed similar results to that of expert dentists	These models need to be explored for disease diagnosis and treatment planning
21	Moran et al. [49]	2021	Cross sectional	CNNs	CNN model (Inception) for identifying approximal DC in bitewing radiographs	112 (45 for testing)	DC lesions	Digital bitewing radiographs	ResNet model	Accuracy of 73.3%	(+)Effective	This model demonstrated promising results in comparison with the reference model	This model can be used for assisting clinicians in decision making
22	Mertens S et al. [50]	2021	Randomized control trial	CNNs	CNN model for detection of proximal DC using bitewing radiographs	140 patients (20 testing)	DC lesions	Bitewing radiographs	5 expert expert dentists	ROC of 0.89 and sensitivity of 0.81 with statistical significance (*p* < 0.05)	(+)Effective	Dentists using AI model demonstrated statistically significant performance in comparison with other dentists	This model can increase diagnostic accuracy of dentists
23	Vinayahalingam et al. [51]	2021	Retrospective cohort	CNNs	To evaluate CNN based model (MobileNet V2) for classifying DC on panoramic radiographs	500 (320 for training, 80 for validating 100 for testing)	DC lesions	Panoramic radiographs	Reference standards	Accuracy of 0.87, sensitivity of 0.86, specificity of 0.88, AUC of 0.90, F1 score of 0.86	(+)Effective	This model displayed good performance in detecting DC in third molars	This model is an initiation for developing a model that can assist clinicians in deciding on removal of third molars
24	Lee et al. [52]	2021	Cross sectional	CNNs	To evaluate deep CNN (U-Net) models for detection of DC in bitewing radiographs	304 for training, 50 for testing	DC lesions	Bitewing radiographs	3 expert dentists	Precision of 63.29%; recall of 65.02%; F1-score of 64.14%	(+)Effective	This model displayed considerable performance in detecting DC	This model can help clinicians in detecting DC more accurately
25	Hur et al. [53]	2021	Retrospective cohort	ANNs	MLmodels for predicting DC on second molars associated with impacted third molars in CBCT and panoramic radiographs	1321 patients (2642 impacted mandibular third molars,1850 for training and 792 for testing)	DC lesions	Panoramic radiographs and CBCT images	Single predictors as reference	ROC of 0.88 to 0.89	(+)Effective	This ML model demonstrated significantly superior performance in prediction of DC in comparison to other models	This model can be of great value for clinicians for preventive treatment and decision making on third molars
26	De Araujo Faria et al. [54]	2021	Retrospective cohort	ANNs	AI based model for prediction and detection of radiation-related caries (RRC) on panoramic radiographs	15 head and neck cancer (HNC) patients	DC lesions	Digital Panoramic radiographs	2 Expert dentists	For detection accuracy of 98.8% AUC = 0.9869 and for prediction and accuracy of 99.2%, AUC = 0.9886	(+)Effective	This model displayed high accuracy in detection and diagnosis of RRC	These models can aid in designing preventive dental care for patients with HNC
27	Wu et al. [55]	2021	Prospective cohort	ANNs	MLmodel identifying caries-related oral microbes in cross-sectional mother-child dyads	37 salivary samples and 36 plaque samples for children DC prediction models. 32 plaque samples for mother DC prediction models.	DC lesions	Data sets	Reference standards	AUC of 0.82 for the child’s saliva model, AUC of 0.78 for the child’s plaque model, and AUC of 0.73 for the mother’s plaque model	(+)Effective	These models achieved desirable results for both mother and children	More variables need to be considered in the future for fine-tuning the models
28	Mao et al. [56]	2021	Cross sectional	CNNs	CNN based model for identifying DC and restorations on bitewing radiographs	278 images (70% for training and 30% for testing)	DC lesions	Bitewing radiographs	Reference models GoogleNet, Vgg19, and ResNet50	Accuracy of 95.56% for restoration judgment and accuracy of 90.30% for DC judgment	(+)Effective	AlexNet model demonstrated high accuracy in comparison to other models	This model can assist dentists in better decision making and treatment planning
29	Park et al. [57]	2021	Prospective cohort	ANNs	ML based AI models (XGBoost, random forest, LightGBM algorithms and Final model) for predicting early childhood caries	4195 (2936 for training and 1259 for testing)	DC lesions	Data sets	Traditional regression model	AUROC = 0.774–0.785	(+)Effective	ML-based models showed favorable performance in predicting DC	Can be useful in identifying high risk groups and implementing preventive treatments
30	Huang et al. [58]	2021	Cross sectional	CNNs	AI based models AlexNet, VGG-16, ResNet-152, Xception, and ResNext-101 for detecting DC	748 cross-sectional 2D images(599 for training and 149 for testing)	DC lesions	OCT and micro-CT images	5 clinicians	ResNet-152 demonstrated highest accuracy rate of 95.21% and sensitivity of 98.85% specificity of 89.83%, and the PPV of 93.48% and NPV was 98.15%,.	(+)Effective	ResNet-152 CNNs models are better than clinicians at distinguishing pathological tooth structures using OCT images	These models can aid clinicians in providing patients with more accurate diagnoses
31	Bayraktar et al. [59]	2022	Cross sectional	CNNs	Assess the performance of CNN based model (YOLO) for diagnosis of interproximal caries lesions on bitewing radiographs	1000 (800 for training and 200 for testing)	DC lesions	Digital bitewing radiographs	2 experienced dentists	Accuracy of 94.59%, sensitivity was 72.26, specificity was 98.19%, PPV was 86.58%, NPV was 95.64% and overall AUC was 87.19%.	(+)Effective	This CNN-based model showed good performance with high accuracy scores	This model can assist clinicians in diagnosing interproximal DC
32	Zhang et al. [60]	2022	Cross sectional	CNNs	To assess the performance of CNN based model (ConvNet) for detecting DC using oral photographs	625 Subjects (3932) oral photographs(2507 for training and 1125 for testing)	DC lesions	Oral photographs	3 board certified dentists	AUC of 85.65%, sensitivity of 81.90%	(+)Effective	The DL model displayed promising results in detecting DC on oral photographs	This is a cost-effective tool for screening of DC
33	Kühnisch et al. [61]	2022	Retrospective cohort	CNNs	To evaluate a (CNNs) based model for detection and categorization of DC using oral photographs	2417 photographs (1891 for training and 479 for testing)	DC lesions	Oral photographs	Expert standards	Accuracy of 92.5%, sensitivity of 89.6; specificity of 94.3; AUC was 0.964	(+)Effective	This DL model displayed promising accuracy in detecting DC using intraoral photographs	This model can be of potential use and feasible in future
34	Zhu et al. [62]	2022	Retrospective cohort	CNNs	A CNNs based model CariesNet to delineate different caries degrees on panoramic radiographs	1159 (900 for training, 135 for validating and 124 for testing)	DC lesions	Panoramic radiographs	Reference models	Mean Dice coefficient of 93.64%, accuracy of 93.61%,F1 score 92.87, precision of 94.09 and recall of 86.01	(+)Effective	This CNN-based model effectively segmented the DC lesions from panoramic radiographs	This model was successful in segmenting even small lesions from large images

ML = machine learning, ANNs = artificial neural networks, CNNs = convolutional neural networks, DCNNs = deep neural networks, c-index = concordance index, CT = computed tomography scans, CBCT = cone-beam computed tomography, OCT = optical coherence tomography.

**Table 3:** Assessment of Strength of Evidence.

Outcome	Strength of Evidence (GRADE)
Application and performance in AI models in prediction of DC [29,33,40,45,46,53,55,56]	⨁⨁⨁◯
Application and performance in AI models in detection and diagnosis of DC [30,31,32,34,35,36,38,39,41,42,43,44,47,48,49,50,52,54,56,58,59,60,61,62]	⨁⨁⨁⨁
Application and performance in classification of DC [37,42]	⨁⨁⨁◯

⨁⨁⨁⨁ Strong Evidence; ⨁⨁⨁◯ Moderate Evidence.

## Data Availability

Not applicable.

## Supplementary Materials

The following supporting information can be downloaded at: https://www.mdpi.com/article/10.3390/diagnostics12051083/s1, Table S1: Assessment of risk of bias domains and applicability concerns.

## References

[1] GBD 2017 Disease and Injury Incidence and Prevalence Collaborators (2018). Global, Regional, and National Incidence, Prevalence, and Years Lived with Disability for 354 Diseases and Injuries for 195 Countries and Territories, 1990–2017: A Systematic Analysis for the Global Burden of Disease Study 2017. Lancet.

[2] Slade G.D., Sanders A.E. (2011). The Paradox of Better Subjective Oral Health in Older Age. J. Dent. Res..

[3] Wen P.Y.F., Chen M.X., Zhong Y.J., Dong Q.Q., Wong H.M. (2021). Global Burden and Inequality of Dental Caries, 1990 to 2019. J. Dent. Res..

[4] Righolt A.J., Jevdjevic M., Marcenes W., Listl S. (2018). Global-, Regional-, and Country-Level Economic Impacts of Dental Diseases in 2015. J. Dent. Res..

[5] Neuhaus K.W., Ellwood R., Lussi A., Pitts N.B. (2009). Traditional Lesion Detection Aids. Monogr. Oral Sci..

[6] Bader J.D., Shugars D.A., Bonito A.J. (2001). Systematic Reviews of Selected Dental Caries Diagnostic and Management Methods. J. Dent. Educ..

[7] Costa A.M., Bezzerra A.C.B., Fuks A.B. (2007). Assessment of the Accuracy of Visual Examination, Bite-Wing Radiographs and DIAGNOdent on the Diagnosis of Occlusal Caries. Eur. Arch. Paediatr. Dent..

[8] Sridhar N., Tandon S., Rao N. (2009). A Comparative Evaluation of DIAGNOdent with Visual and Radiography for Detection of Occlusal Caries: An in Vitro Study. Indian J. Dent. Res..

[9] Schneiderman A., Elbaum M., Shultz T., Keem S., Greenebaum M., Driller J. (1997). Assessment of Dental Caries with Digital Imaging Fiber-Optic TransIllumination (DIFOTI): In Vitro Study. Caries Res..

[10] El-Housseiny A.A., Jamjoum H. (2001). Evaluation of Visual, Explorer, and a Laser Device for Detection of Early Occlusal Caries. J. Clin. Pediatr. Dent..

[11] Vaarkamp J., ten Bosch J.J., Verdonschot E.H., Bronkhoorst E.M. (2000). The Real Performance of Bitewing Radiography and Fiber-Optic Transillumination in Approximal Caries Diagnosis. J. Dent. Res..

[12] Matalon S., Feuerstein O., Kaffe I. (2003). Diagnosis of Approximal Caries: Bite-Wing Radiology versus the Ultrasound Caries Detector. An in Vitro Study. Oral Surg. Oral Med. Oral Pathol. Oral Radiol. Endod..

[13] Cagetti M.G., Bontà G., Cocco F., Lingstrom P., Strohmenger L., Campus G. (2018). Are Standardized Caries Risk Assessment Models Effective in Assessing Actual Caries Status and Future Caries Increment? A Systematic Review. BMC Oral Health.

[14] Medeiros F.A., Jammal A.A., Thompson A.C. (2019). From Machine to Machine: An OCT-Trained Deep Learning Algorithm for Objective Quantification of Glaucomatous Damage in Fundus Photographs. Ophthalmology.

[15] Zheng X., Yao Z., Huang Y., Yu Y., Wang Y., Liu Y., Mao R., Li F., Xiao Y., Wang Y. (2020). Deep Learning Radiomics Can Predict Axillary Lymph Node Status in Early-Stage Breast Cancer. Nat. Commun..

[16] Esteva A., Kuprel B., Novoa R.A., Ko J., Swetter S.M., Blau H.M., Thrun S. (2017). Dermatologist-Level Classification of Skin Cancer with Deep Neural Networks. Nature.

[17] Li R., Xiao C., Huang Y., Hassan H., Huang B. (2022). Deep Learning Applications in Computed Tomography Images for Pulmonary Nodule Detection and Diagnosis: A Review. Diagnostics.

[18] Hassan H., Ren Z., Zhao H., Huang S., Li D., Xiang S., Kang Y., Chen S., Huang B. (2022). Review and Classification of AI-Enabled COVID-19 CT Imaging Models Based on Computer Vision Tasks. Comput. Biol. Med..

[19] Chang H.-J., Lee S.-J., Yong T.-H., Shin N.-Y., Jang B.-G., Kim J.-E., Huh K.-H., Lee S.-S., Heo M.-S., Choi S.-C. (2020). Deep Learning Hybrid Method to Automatically Diagnose Periodontal Bone Loss and Stage Periodontitis. Sci. Rep..

[20] Yang H., Jo E., Kim H.J., Cha I.-H., Jung Y.-S., Nam W., Kim J.-Y., Kim J.-K., Kim Y.H., Oh T.G. (2020). Deep Learning for Automated Detection of Cyst and Tumors of the Jaw in Panoramic Radiographs. J. Clin. Med..

[21] Kwon O., Yong T.-H., Kang S.-R., Kim J.-E., Huh K.-H., Heo M.-S., Lee S.-S., Choi S.-C., Yi W.-J. (2020). Automatic Diagnosis for Cysts and Tumors of Both Jaws on Panoramic Radiographs Using a Deep Convolution Neural Network. Dentomaxillofac. Radiol..

[22] Hung K., Montalvao C., Tanaka R., Kawai T., Bornstein M.M. (2020). The Use and Performance of Artificial Intelligence Applications in Dental and Maxillofacial Radiology: A Systematic Review. Dentomaxillofac. Radiol..

[23] Bichu Y.M., Hansa I., Bichu A.Y., Premjani P., Flores-Mir C., Vaid N.R. (2021). Applications of Artificial Intelligence and Machine Learning in Orthodontics: A Scoping Review. Prog. Orthod..

[24] Bernauer S.A., Zitzmann N.U., Joda T. (2021). The Use and Performance of Artificial Intelligence in Prosthodontics: A Systematic Review. Sensors.

[25] Revilla-León M., Gómez-Polo M., Vyas S., Barmak B.A., Özcan M., Att W., Krishnamurthy V.R. (2021). Artificial Intelligence Applications in Restorative Dentistry: A Systematic Review. J. Prosthet. Dent..

[26] Schwendicke F., Rossi J.G., Göstemeyer G., Elhennawy K., Cantu A.G., Gaudin R., Chaurasia A., Gehrung S., Krois J. (2021). Cost-Effectiveness of Artificial Intelligence for Proximal Caries Detection. J. Dent. Res..

[27] McGrath T.A., Alabousi M., Skidmore B., Korevaar D.A., Bossuyt P.M.M., Moher D., Thombs B., McInnes M.D.F. (2017). Recommendations for Reporting of Systematic Reviews and Meta-Analyses of Diagnostic Test Accuracy: A Systematic Review. Syst. Rev..

[28] Whiting P.F., Rutjes A.W.S., Westwood M.E., Mallett S., Deeks J.J., Reitsma J.B., Leeflang M.M.G., Sterne J.A.C., Bossuyt P.M.M., QUADAS-2 Group (2011). QUADAS-2: A Revised Tool for the Quality Assessment of Diagnostic Accuracy Studies. Ann. Intern. Med..

[29] Zanella-Calzada L., Galván-Tejada C., Chávez-Lamas N., Rivas-Gutierrez J., Magallanes-Quintanar R., Celaya-Padilla J., Galván-Tejada J., Gamboa-Rosales H. (2018). Deep Artificial Neural Networks for the Diagnostic of Caries Using Socioeconomic and Nutritional Features as Determinants: Data from NHANES 2013–2014. Bioengineering.

[30] Lee J.-H., Kim D.-H., Jeong S.-N., Choi S.-H. (2018). Detection and Diagnosis of Dental Caries Using a Deep Learning-Based Convolutional Neural Network Algorithm. J. Dent..

[31] Choi J., Eun H., Kim C. (2018). Boosting Proximal Dental Caries Detection via Combination of Variational Methods and Convolutional Neural Network. J. Signal Process. Syst..

[32] Casalegno F., Newton T., Daher R., Abdelaziz M., Lodi-Rizzini A., Schürmann F., Krejci I., Markram H. (2019). Caries Detection with Near-Infrared Transillumination Using Deep Learning. J. Dent. Res..

[33] Hung M., Voss M.W., Rosales M.N., Li W., Su W., Xu J., Bounsanga J., Ruiz-Negrón B., Lauren E., Licari F.W. (2019). Application of Machine Learning for Diagnostic Prediction of Root Caries. Gerodontology.

[34] Cantu A.G., Gehrung S., Krois J., Chaurasia A., Rossi J.G., Gaudin R., Elhennawy K., Schwendicke F. (2020). Detecting Caries Lesions of Different Radiographic Extension on Bitewings Using Deep Learning. J. Dent..

[35] Geetha V., Aprameya K.S., Hinduja D.M. (2020). Dental Caries Diagnosis in Digital Radiographs Using Back-Propagation Neural Network. Health Inf. Sci. Syst..

[36] Schwendicke F., Elhennawy K., Paris S., Friebertshäuser P., Krois J. (2020). Deep Learning for Caries Lesion Detection in Near-Infrared Light Transillumination Images: A Pilot Study. J. Dent..

[37] Karhade D.S., Roach J., Shrestha P., Simancas-Pallares M.A., Ginnis J., Burk Z.J.S., Ribeiro A.A., Cho H., Wu D., Divaris K. (2021). An Automated Machine Learning Classifier for Early Childhood Caries. Pediatr. Dent..

[38] Duong D.L., Kabir M.H., Kuo R.F. (2021). Automated Caries Detection with Smartphone Color Photography Using Machine Learning. Health Inform. J..

[39] Duong D.L., Nguyen Q.D.N., Tong M.S., Vu M.T., Lim J.D., Kuo R.F. (2021). Proof-of-Concept Study on an Automatic Computational System in Detecting and Classifying Occlusal Caries Lesions from Smartphone Color Images of Unrestored Extracted Teeth. Diagnostics.

[40] Ramos-Gomez F., Marcus M., Maida C.A., Wang Y., Kinsler J.J., Xiong D., Lee S.Y., Hays R.D., Shen J., Crall J.J. (2021). Using a Machine Learning Algorithm to Predict the Likelihood of Presence of Dental Caries among Children Aged 2 to 7. Dent. J..

[41] Askar H., Krois J., Rohrer C., Mertens S., Elhennawy K., Ottolenghi L., Mazur M., Paris S., Schwendicke F. (2021). Detecting White Spot Lesions on Dental Photography Using Deep Learning: A Pilot Study. J. Dent..

[42] Chen H., Li H., Zhao Y., Zhao J., Wang Y. (2021). Dental Disease Detection on Periapical Radiographs Based on Deep Convolutional Neural Networks. Int. J. Comput. Assist. Radiol. Surg..

[43] Devlin H., Williams T., Graham J., Ashley M. (2021). The ADEPT Study: A Comparative Study of Dentists’ Ability to Detect Enamel-Only Proximal Caries in Bitewing Radiographs with and without the Use of Assist Dent Artificial Intelligence Software. Br. Dent. J..

[44] Bayrakdar I.S., Orhan K., Akarsu S., Çelik Ö., Atasoy S., Pekince A., Yasa Y., Bilgir E., Sağlam H., Aslan A.F. (2021). Deep-Learning Approach for Caries Detection and Segmentation on Dental Bitewing Radiographs. Oral Radiol..

[45] Zaorska K., Szczapa T., Borysewicz-Lewicka M., Nowicki M., Gerreth K. (2021). Prediction of Early Childhood Caries Based on Single Nucleotide Polymorphisms Using Neural Networks. Genes.

[46] Pang L., Wang K., Tao Y., Zhi Q., Zhang J., Lin H. (2021). A New Model for Caries Risk Prediction in Teenagers Using a Machine Learning Algorithm Based on Environmental and Genetic Factors. Front. Genet..

[47] Zheng L., Wang H., Mei L., Chen Q., Zhang Y., Zhang H. (2021). Artificial Intelligence in Digital Cariology: A New Tool for the Diagnosis of Deep Caries and Pulpitis Using Convolutional Neural Networks. Ann. Transl. Med..

[48] Lian L., Zhu T., Zhu F., Zhu H. (2021). Deep Learning for Caries Detection and Classification. Diagnostics.

[49] Moran M., Faria M., Giraldi G., Bastos L., Oliveira L., Conci A. (2021). Classification of Approximal Caries in Bitewing Radiographs Using Convolutional Neural Networks. Sensors.

[50] Mertens S., Krois J., Cantu A.G., Arsiwala L.T., Schwendicke F. (2021). Artificial Intelligence for Caries Detection: Randomized Trial. J. Dent..

[51] Vinayahalingam S., Kempers S., Limon L., Deibel D., Maal T., Hanisch M., Bergé S., Xi T. (2021). Classification of Caries in Third Molars on Panoramic Radiographs Using Deep Learning. Sci. Rep..

[52] Lee S., Oh S.-I., Jo J., Kang S., Shin Y., Park J.-W. (2021). Deep Learning for Early Dental Caries Detection in Bitewing Radiographs. Sci. Rep..

[53] Hur S.-H., Lee E.-Y., Kim M.-K., Kim S., Kang J.-Y., Lim J.S. (2021). Machine Learning to Predict Distal Caries in Mandibular Second Molars Associated with Impacted Third Molars. Sci. Rep..

[54] De Araujo Faria V., Azimbagirad M., Viani Arruda G., Fernandes Pavoni J., Cezar Felipe J., Dos Santos E.M.C.M.F., Murta Junior L.O. (2021). Prediction of Radiation-Related Dental Caries through PyRadiomics Features and Artificial Neural Network on Panoramic Radiography. J. Digit. Imaging.

[55] Wu T.T., Xiao J., Sohn M.B., Fiscella K.A., Gilbert C., Grier A., Gill A.L., Gill S.R. (2021). Machine Learning Approach Identified Multi-Platform Factors for Caries Prediction in Child-Mother Dyads. Front. Cell. Infect. Microbiol..

[56] Mao Y.-C., Chen T.-Y., Chou H.-S., Lin S.-Y., Liu S.-Y., Chen Y.-A., Liu Y.-L., Chen C.-A., Huang Y.-C., Chen S.-L. (2021). Caries and Restoration Detection Using Bitewing Film Based on Transfer Learning with CNNs. Sensors.

[57] Park Y.-H., Kim S.-H., Choi Y.-Y. (2021). Prediction Models of Early Childhood Caries Based on Machine Learning Algorithms. Int. J. Environ. Res. Public Health.

[58] Huang Y.-P., Lee S.-Y. (2021). Deep Learning for Caries Detection Using Optical Coherence Tomography. bioRxiv.

[59] Bayraktar Y., Ayan E. (2022). Diagnosis of Interproximal Caries Lesions with Deep Convolutional Neural Network in Digital Bitewing Radiographs. Clin. Oral Investig..

[60] Zhang X., Liang Y., Li W., Liu C., Gu D., Sun W., Miao L. (2022). Development and Evaluation of Deep Learning for Screening Dental Caries from Oral Photographs. Oral Dis..

[61] Kühnisch J., Meyer O., Hesenius M., Hickel R., Gruhn V. (2022). Caries Detection on Intraoral Images Using Artificial Intelligence. J. Dent. Res..

[62] Zhu H., Cao Z., Lian L., Ye G., Gao H., Wu J. (2022). CariesNet: A Deep Learning Approach for Segmentation of Multi-Stage Caries Lesion from Oral Panoramic X-ray Image. Neural Comput. Appl..

[63] Granholm A., Alhazzani W., Møller M.H. (2019). Use of the GRADE Approach in Systematic Reviews and Guidelines. Br. J. Anaesth..

[64] Bader J.D., Shugars D.A., Bonito A.J. (2002). A Systematic Review of the Performance of Methods for Identifying Carious Lesions. J. Public Health Dent..

[65] Geibel M.-A., Carstens S., Braisch U., Rahman A., Herz M., Jablonski-Momeni A. (2017). Radiographic Diagnosis of Proximal Caries-Influence of Experience and Gender of the Dental Staff. Clin. Oral Investig..

[66] Tinanoff N., Baez R.J., Diaz Guillory C., Donly K.J., Feldens C.A., McGrath C., Phantumvanit P., Pitts N.B., Seow W.K., Sharkov N. (2019). Early Childhood Caries Epidemiology, Aetiology, Risk Assessment, Societal Burden, Management, Education, and Policy: Global Perspective. Int. J. Paediatr. Dent..

[67] He K., Zhang X., Ren S., Sun J. Deep Residual Learning for Image Recognition. Proceedings of the IEEE Conference on Computer Vision and Pattern Recognition.

[68] Sabour S., Frosst N., Hinton G.E. (2017). Dynamic Routing between Capsules. Adv. Neural Inf. Processing Syst..

[69] Yang V., Zhu Y., Curtis D., Le O., Chang N.Y.N., Fried W.A., Simon J.C., Banan P., Darling C.L., Fried D. (2020). Thermal Imaging of Root Caries In Vivo. J. Dent. Res..

