# Supplementary material for: Application and Performance of Artificial Intelligence Technology in Detection, Diagnosis and Prediction of Dental Caries (DC)—A Systematic Review
Source: Diagnostics (Basel). 2022 Apr 26;12(5):1083. doi: 10.3390/diagnostics12051083 (PMC9139989; doi:10.3390/diagnostics12051083)
Supplement: Supplementary file 1 [file diagnostics-12-01083-s001.zip › diagnostics-1666673-supplementary.pdf]

Table S1: Assessment of risk of bias domains and applicability concerns

Study	RISK OF BIAS				APPLICABILITY CONCERNS		
	PATIENT SELECTION	INDEX TEST	REFERENCE STANDARD	FLOW AND TIMING	PATIENT SELECTION	INDEX TEST	REFERENCE STANDARD
Zanella-Calzada LA et al.[23]	low Risk	low Risk	low Risk	low Risk	low Risk	low Risk	low Risk
Lee JH et al.[24]	low Risk	low Risk	low Risk	Unclear	low Risk	low Risk	Unclear
Choi J et al.[25]	low Risk	Unclear	high risk	high risk	low Risk	Unclear	high risk
Casalegno F et al.[26]	Unclear	high risk	Unclear	low Risk	Unclear	high risk	high risk
Hung M et al.[27]	low Risk	low Risk	low Risk	low Risk	low Risk	low Risk	low Risk
Cantu AG et al.[28]	low Risk	low Risk	high risk	low Risk	low Risk	high risk	Unclear
Geetha V et al.[29]	high risk	low Risk	Unclear	low Risk	low Risk	low Risk	low Risk
Schwendicke F et al.[30]	low Risk	low Risk	low Risk	low Risk	low Risk	low Risk	low Risk
Karhade D.S et al.[31]	Unclear	high risk	low Risk	Unclear	Unclear	low Risk	low Risk
Duong DL et al.[32]	low Risk	low Risk	low Risk	low Risk	low Risk	low Risk	low Risk
Duong DL et al.[33]	low Risk	low Risk	low Risk	low Risk	low Risk	low Risk	low Risk
Ramos-Gomez F et al.[34]	low Risk	low Risk	low Risk	low Risk	high risk	low Risk	Unclear
Askar H et al.[35]	high risk	low Risk	low Risk	low Risk	high risk	Unclear	low Risk
Chen H et al.[36]	low Risk	low Risk	low Risk	Unclear	low Risk	high risk	low Risk
Devlin H et al.[37]	low Risk	low Risk	low Risk	low Risk	low Risk	low Risk	low Risk
Bayrakdar IS et al.[38]	low Risk	low Risk	low Risk	low Risk	low Risk	low Risk	low Risk
Zaorska K et al.[39]	low Risk	low Risk	high risk	Unclear	low Risk	low Risk	low Risk
Pang L et al.[40]	low Risk	low Risk	high risk	low Risk	Unclear	Unclear	low Risk
Zheng L et al[41]	low Risk	low Risk	Unclear	low Risk	low Risk	low Risk	low Risk
Lian L et al[42]	low Risk	low Risk	high risk	Unclear	low Risk	low Risk	low Risk
Moran M et al[43]	Unclear	low Risk	low Risk	low Risk	Unclear	low Risk	low Risk
Mertens S et al.[44]	low Risk	low Risk	low Risk	low Risk	low Risk	low Risk	low Risk
Vinayahalingam S et al[45]	high risk	Unclear	high risk	Unclear	high risk	Unclear	Unclear
Lee S et al[46]	high risk	low Risk	high risk	Unclear	high risk	high risk	high risk
Hur SH et al[47]	low Risk	high risk	high risk	Unclear	high risk	low Risk	low Risk
De Araujo Faria V et al[48]	low Risk	low Risk	low Risk	low Risk	low Risk	low Risk	low Risk
Wu TT et al[49]	low Risk	low Risk	low Risk	low Risk	low Risk	low Risk	low Risk
Mao YC et al[50]	Unclear	low Risk	Unclear	Unclear	Unclear	low Risk	low Risk
Park YH et al[51]	low Risk	low Risk	Unclear	Unclear	low Risk	low Risk	low Risk
YP Huang et al[52]	low Risk	low Risk	low Risk	low Risk	low Risk	low Risk	low Risk
Bayraktar Y et al[53]	low Risk	low Risk	low Risk	low Risk	low Risk	low Risk	low Risk
Zhang X et al[54]	low Risk	low Risk	low Risk	low Risk	low Risk	low Risk	low Risk
Kühnisch J et al[55]	low Risk	low Risk	low Risk	low Risk	low Risk	low Risk	low Risk
Zhu H et al[56]	low Risk	low Risk	Unclear	low Risk	low Risk	low Risk	Unclear
